# The complete chloroplast genome sequence of *Casimiroa edulis*

**DOI:** 10.1080/23802359.2019.1688711

**Published:** 2019-11-12

**Authors:** Dejun Yang, Qiong Qiu, Linhong Xu, Yumei Xu, Yi Wang

**Affiliations:** a Institute of Tropical Forestry, Yunnan Academy of Forestry, Kunming, People’s Republic of China;; b Laboratory of Forest Plant Cultivation and Utilization, Yunnan Academy of Forestry, Kunming, People’s Republic of China

**Keywords:** *Casimiroa edulis*, chloroplast, Illumina sequencing, phylogenetic analysis

## Abstract

The first complete chloroplast genome (cpDNA) sequence of *Casimiroa edulis* was determined from Illumina HiSeq pair-end sequencing data in this study. The cpDNA is 160,176 bp in length, contains a large single-copy region (LSC) of 87,536 bp and a small single-copy region (SSC) of 18,576 bp, which were separated by a pair of inverted repeats (IR) regions of 27,032 bp. The genome contains 131 genes, including 86 protein-coding genes, 8 ribosomal RNA genes, and 37 transfer RNA genes. The overall GC content of the whole genome is 38.2%, and the corresponding values of the LSC, SSC, and IR regions are 36.5, 33.0, and 42.9%, respectively. Further, phylogenomic analysis showed that *C. edulis, Phellodendron amurense*, and *Zanthoxylum bungeanum* clustered in a clade in family Rutaceae.

*Casimiroa edulis* is the species of the genus *Casimiroa* within the family Rutaceae and is widely distributed in the tropical and subtropical regions of Mesoamerica including Mexico (Ibrahim et al. 2019), it is an evergreen tree that reaches up to 18 m and is of great value in economy as it produces an edible fruit popularly known as white sapote (Agustin et al. 2017). With the sweet flavor and medicinal properties, *C. edulis* has been cultivated around the world (Sohn and Epstein 2019). The extracts of *C. edulis* showed anticonvulsant, antihypertensive activities (Magos et al. 1998). *Casimiroa edulis* also was used as sedative, hypnotic in Mexico (Awaad et al. 2012). However, there have been no genomic studies on *C. edulis*.

Herein, we reported and characterized the complete *C. edulis* plastid genome (MN539263). One *C. edulis* individual (specimen number: 201807055) was collected from Puwen, Yunnan Province of China (23°34′11′′N, 101°17′12′′E). The specimen is stored at Yunnan Academy of Forestry Herbarium, Kunming, China and the accession number is YAFH0012757. DNA was extracted from its fresh leaves using DNA Plantzol Reagent (Invitrogen, Carlsbad, CA, USA).

Paired-end reads were sequenced by using Illumina HiSeq system (Illumina, San Diego, CA). In total, about 25.1 million high-quality clean reads were generated with adaptors trimmed. Aligning, assembly, and annotation were conducted by CLC de novo assembler (CLC Bio, Aarhus, Denmark), BLAST, GeSeq (Tillich et al. 2017), and GENEIOUS version 11.0.5 (Biomatters Ltd, Auckland, New Zealand). To confirm the phylogenetic position of *C. edulis*, other nine species of family *Rutaceae* from NCBI were aligned using MAFFT version 7 (Katoh and Standley 2013). The Auto algorithm in the MAFFT alignment software was used to align the eight complete genome sequences and the G-INS-i algorithm was used to align the partial complex sequecnces and maximum likelihood (ML) bootstrap analysis was conducted using RAxML (Stamatakis 2006); bootstrap probability values were calculated from 1000 replicates. *Swietenia macrophylla* (MH348156) and *Khaya senegalensis* (KX364458) were served as the out-group.

The complete *C. edulis* plastid genome is a circular DNA molecule with the length of 160,176 bp, contains a large single-copy region (LSC) of 87,536 bp and a small single-copy region (SSC) of 18,576 bp, which were separated by a pair of inverted repeats (IR) regions of 27,032 bp. The overall GC content of the whole genome is 38.2%, and the corresponding values of the LSC, SSC, and IR regions are 36.5, 33.0, and 42.9%, respectively. The plastid genome contained 131 genes, including 86 protein-coding genes, 8 ribosomal RNA genes, and 37 transfer RNA genes. Phylogenetic analysis showed that *C. edulis, Phellodendron amurense*, and *Zanthoxylum bungeanum* clustered in a clade in family *Rutaceae* (Figure 1). The determination of the complete plastid genome sequences provided new molecular data to illuminate the *Rutaceae* evolution.

**Figure 1.**
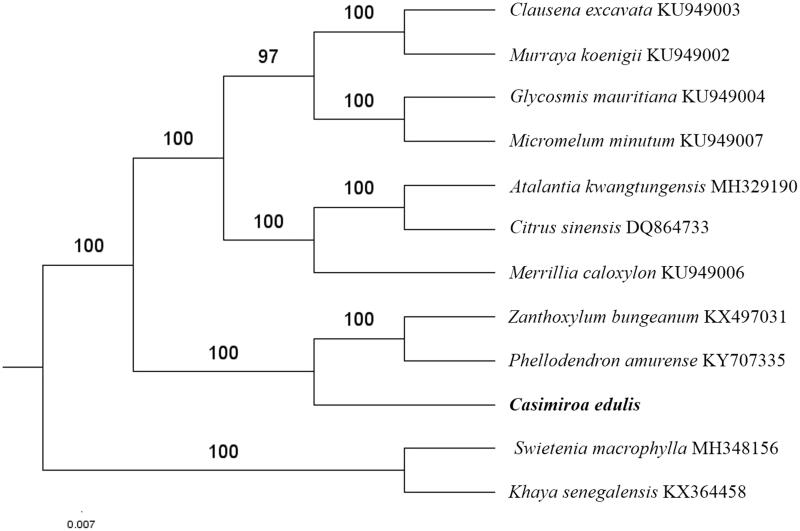
The maximum-likelihood tree based on the 10 chloroplast genomes of *Rutaceae*. The bootstrap value based on 1000 replicates is shown on each node.
